# Telomere Length Attrition, a Marker of Biological Senescence, Is Inversely Correlated with Triglycerides and Cholesterol in South Asian Males with Type 2 Diabetes Mellitus

**DOI:** 10.1155/2012/895185

**Published:** 2012-03-01

**Authors:** Alison L. Harte, Nancy F. da Silva, Michelle A. Miller, Francesco P. Cappuccio, Ann Kelly, Joseph P. O'Hare, Anthony H. Barnett, Nasser M. Al-Daghri, Omar Al-Attas, Majed Alokail, Shaun Sabico, Gyanendra Tripathi, Srikanth Bellary, Sudhesh Kumar, Philip G. McTernan

**Affiliations:** ^1^Clinical Sciences Research Laboratories, Division of Metabolic and Vascular Health, Warwick Medical School, University of Warwick, University Hospital Coventry and Warwickshire, Coventry CV2 2DX, UK; ^2^AstraZeneca R&D, CVGI Bioscience, Mereside, Alderley Park, Macclesfield, Chesire SK1 04TG, UK; ^3^University of Birmingham and Biomedical Research Centre, Heart of England NHS Foundation Trust, Birmingham B4 7ET, UK; ^4^Biomarkers Research Program, Biochemistry Department, College of Science, King Saud University, Riyadh 11451, Saudi Arabia; ^5^Center of Excellence in Biotechnology, King Saud University, Riyadh 11451, Saudi Arabia; ^6^Aston University, Birmingham B4 7ET, UK

## Abstract

South Asians have a higher risk of type 2 diabetes mellitus (T2DM) and cardiovascular disease (CVD) than white Caucasians, for a given BMI. Premature biological ageing, assessed by reduction in telomere length (TL), may be mediated by factors resulting from altered metabolic profiles associated with obesity. We hypothesise that ethnicity and metabolic status represent detrimental factors contributing to premature biological ageing. Therefore we assessed TL in two South Asian, age and BMI-matched cohorts [T2DM (*n* = 142) versus non-T2DM (*n* = 76)] to determine the effects of BMI, gender, lipid and CVD profile on biological ageing. Genomic DNA was obtained from the UKADS cohort; biochemical and anthropometric data was collected and TL was measured by quantitative real-time PCR. Our findings indicated a gender-specific effect with reduced TL in T2DM men compared with non-T2DM men (*P* = 0.006). Additionally, in T2DM men, TL was inversely correlated with triglycerides and total cholesterol (*r* = −0.419, *P* < 0.01; *r* = −0.443, *P* < 0.01). In summary, TL was reduced amongst South Asian T2DM men and correlated with triglycerides and total cholesterol. This study highlights enhanced biological ageing among South Asian, T2DM men, which appears to be tracked by changes in lipids and BMI, suggesting that raised lipids and BMI may directly contribute to premature ageing.

## 1. Introduction

People of South Asian origin have a higher predisposition to the development of obesity-related diseases, including type 2 diabetes mellitus (T2DM) when compared with white Caucasians and other ethnicities [1, 2]. Increased metabolic risk among South Asians has been linked to significantly higher leptin levels when compared to other ethnic groups and leptin is a predictor for cardiovascular risk and T2DM. Heightened leptin levels in South Asians may be due to the higher body fat percentage, with more pronounced abdominal obesity compared with other ethnic groups matched for the same body mass index (BMI). This unfavourable body composition promotes decreased insulin sensitivity in this racial group [3]. Dyslipidaemia, specifically low high-density lipoprotein cholesterol (HDL-cholesterol), is also more common among South Asians compared with Europeans and East Asians [4]. These differences in metabolic phenotypes shed light on ethnic discrepancies in risk assessment and support the need for customized screening and diagnosis to optimize treatment. We have previously demonstrated that culturally tailored diabetes care among South Asians leads to enhanced health outcomes as compared to conventional standard care [5].

Telomere length (TL) may play an important role in understanding premature ageing due to the influences of metabolic parameters across ethnic groups. Telomeres are tandem repeats of DNA sequences (TTAGGG) at the end of chromosomes that play an important part in the biology of eukaryotic cells [6], a role as biomarkers of senescence and ageing has been established in many studies, with TL decreasing with advancing age [7–9]. Furthermore, ethnic differences in TL have been observed in different ethnic groups, with black Afro-Caribbean and Hispanic ethnic groups showing greater differences in age-associated TL than white Caucasians [10]. TL has also been associated with many modifiable factors such as lifestyle and marital status [11, 12]. More importantly, there is a significant influence of age-related diseases such as coronary heart disease (CHD), T2DM and cancer on TL reduction [13–16]. 

Several studies confirm the presence of shorter TL among various ethnicities in patients with T2DM and insulin resistance [15, 17]. To our knowledge, no study has reported data on South Asians with respect to TL and metabolic state.

Our cross-sectional study aims to determine whether premature biological ageing, assessed by reduction in TL, is evident in a cohort phenotypically predisposed to develop insulin resistance. Specifically, we aim to identify associations between TL and BMI, gender, lipid, and cardiovascular profile in both T2DM and non-T2DM South Asians.

## 2. Materials and Methods

### 2.1. Subjects

Age- and BMI-matched T2DM and subjects, aged between 45–60 years old, were randomly selected from the UK Asian Diabetes Study (UKADS, T2DM) [18, 19] and from the Wandsworth Heart and Stroke Study (WHSS, non-T2DM) [20, 21]. The selection of the age range was utilized to ensure sufficient power for the study and based on the UK Prospective Diabetes Study (UKPDS) population matching the non-T2DM cohort. Biochemical and anthropometric data were collected for T2DM and non-T2DM subjects, which are detailed in tables 1 and 2. Routine T2DM management, dietary intervention, first line therapy (metformin and combination fibrates), as deemed clinically appropriate, were implemented.

### 2.2. UKADS and WHSS Plasma and Genomic DNA Extraction

Venous blood (2 × 9 mL) per subject was collected in EDTA-coated vacutainers and allowed to stand upright at 4°C overnight. Plasma (1 mL) was removed from each tube and stored at −80°C until required. The remaining blood samples were used for DNA extraction. Genomic DNA was extracted using an adaptation of the Nucleon BACC2 protocol (GE Healthcare, Little Chalfont, Buckinghamshire, UK). The DNA was resuspended in 200 *μ*L of TE buffer (10 mM Tris, 0.1 mM EDTA, pH 8) and quantified by fluorometry using PicoGreen dye (Invitrogen-Molecular Probes, Paisley, UK) as well as by spectrophotometry, using the Nanodrop ND-1000 (NanoDrop Technologies, Wilmington, DE, USA).

### 2.3. TL Measurement

 TL was measured by quantitative real-time polymerase chain reaction (RT-PCR) using the iQCycler real-time PCR system (Bio-Rad Laboratories, Hercules, CA, USA) [22]. In brief, TL was measured using the following telomere primers: primer Tel1b (5′CGGTTTGTTTGGGTTTGGGTTTGGGTTTGGGTTTGGGTT 3′, 450 nM), Tel2b (5′GGCTTGCCTACCCTTACCCTTACCCTTACCCTTACCCT 3′, 450 nM primer), 10 ng genomic DNA, 2xiQ SYBR Green Supermix (Bio-Rad Laboratories, Hercules, CA, USA) in a 30 *μ*L reaction. The reactions were set up in quadruplicate in 96-well plates non-T2DM subjects. For each plate four DNA quantity standards (serial dilution ranging from 15 and 1.87 ng per 30 *μ*L reaction) and three TL standards were also analysed. The three TL standards included 15 ng of genomic DNA from isogenic human thyroid-derived cell lines: K1E72 (TL 3110 bp) and K1E71 (TL 3440 bp) and from a human lung fibroblast cell line MRC5 (TL 8470 bp). Standard curves were derived to convert the threshold cycle number (ΔCT) into starting quantity (SQ) of DNA and to convert the telomere/single copy gene SQ ratio to TL in base pairs (bp) [21–23]. The PCR cycle conditions were as follows: 95°C for 3 min, 40 cycles of 95°C for 15 s and 56°C for 60 s. In order to confirm the PCR specificity, a melting curve (dissociation curve) was performed by 46 repeats of increasing temperature, as previously described [22]. TL is expressed as mean ± standard deviation (SD).

### 2.4. Measurement of Insulin, Glucose, and Lipid Profile Levels

Plasma samples were analyzed for determination of insulin protein concentrations by a solid phase enzyme linked immunosorbant assay (Linco Research Inc. Missouri, USA; Insulin CV intraassay 5.96%, interassay 10.3 ± 0.9%). Glucose concentration was determined by a glucose oxidase method (YSI 200 STAT Plus, YSI Incorporated, Yellow Springs, OH, USA). Lipid profile was measured by the Clinical Biochemistry Laboratory at the University of Birmingham/Heartlands/Queen Elizabeth Hospitals.

### 2.5. Statistical Analysis

Linear regressions, graphical functions, intergroup comparisons and Pearson's correlations were performed using the Statistical Package for the Social Sciences software (SPSS version 14.0 SPSS Inc., Chicago, IL, USA). TL and triglyceride data displayed a non-Gaussian distribution; therefore both data sets were accordingly log transformed prior to analysis. TL is represented by mean ± SD and adjusted for age.

## 3. Results

**Figure 1:**
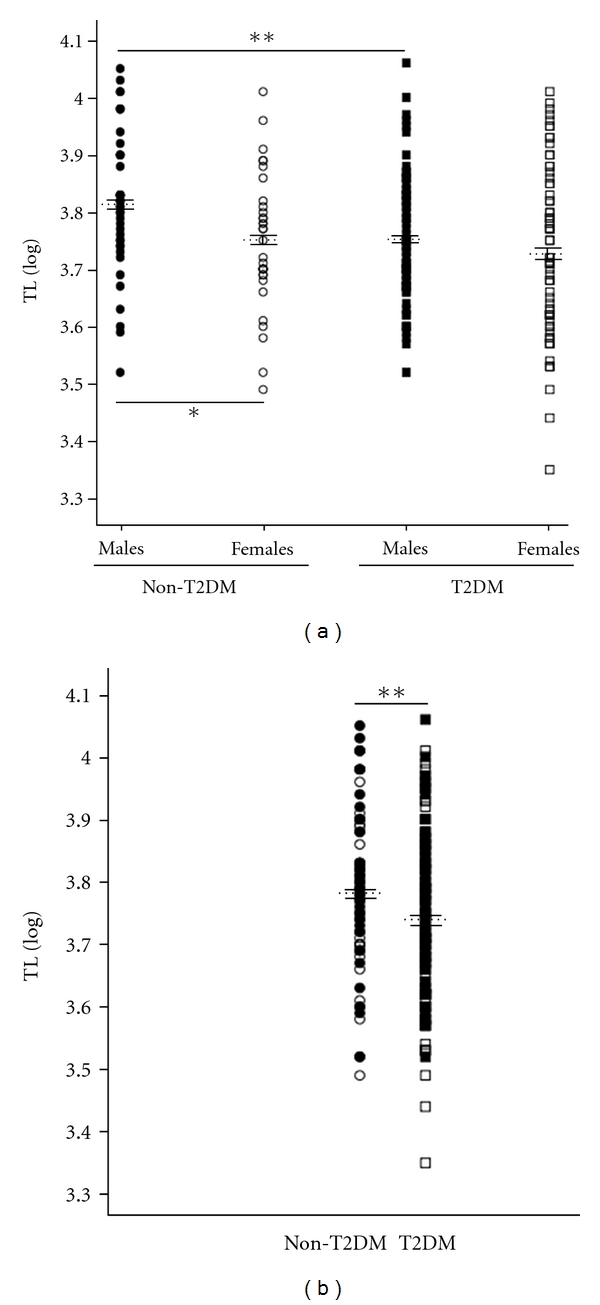
(a) Gender distribution of telomere length (TL) in peripheral blood mononuclear cells in South Asian non-T2DM (male: dark circles, females: open circles) and T2DM (male: dark squares, females: open squares); (b) TL in peripheral blood mononuclear cells in South Asian non-T2DM (circles) and T2DM (squares). TL is represented as mean (dotted line) ± SD, *P < 0.05 and **P < 0.01.

*TL in subjects with T2DM. *The general characteristics of both groups were presented in Tables 1 and 2. Mean TL for T2DM South Asian subjects was 5811.03 ± 1945.52 bp (log⁡^10^ TL: 3.74 ± 0.13). We also examined TL for gender differences and noted that males had a mean TL of 6040.64 ± 2004,37 bp (log⁡^10^ TL: 3.76 ± 0.11) while females had 5608.61 ± 1882.12 bp (log⁡^10^ TL : 3.73 ± 0.14). TL did not display gender dimorphism in T2DM South Asian subjects (*P* = NS). Age and BMI adjusted comparisons revealed that male T2DM subjects had significantly reduced TL compared with their non-T2DM counterparts (*P* = 0.008 (log⁡^10^ TL), Figure 1(a)). This difference was not observed in the female cohort. Mean TL of T2DM was significantly reduced as compared with non-T2DM South Asians (*P* = 0.009 (log⁡^10^ TL), (Figure 1(b)).

### 3.1. Lipid Profile and TL in South Asian T2DM Subjects

**Figure 2:**
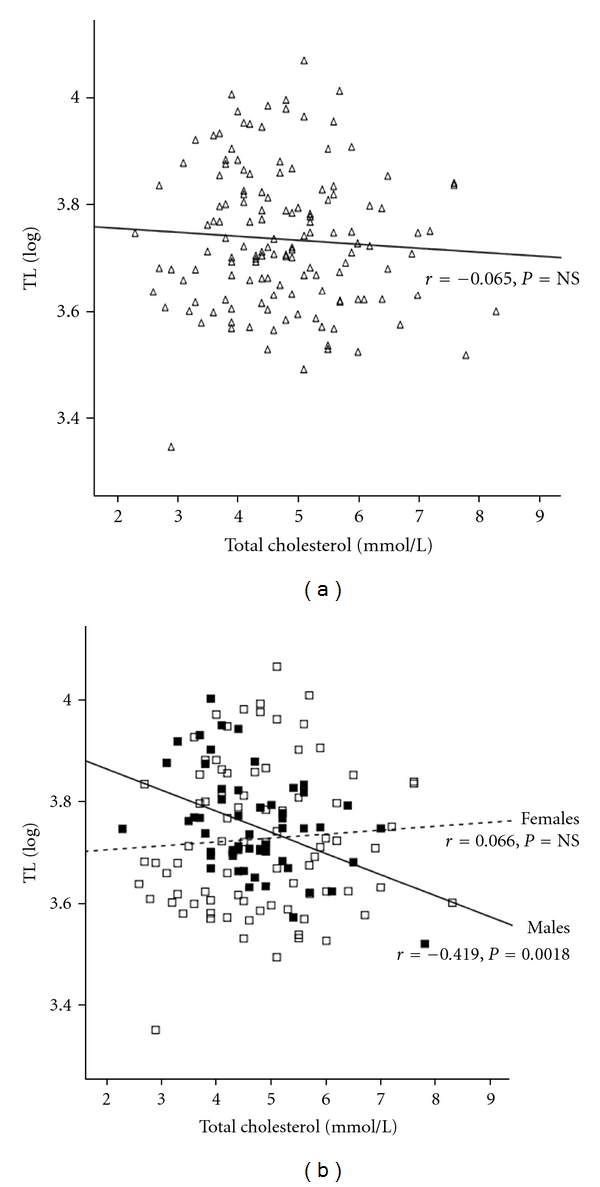
The relationship between total cholesterol and TL in South Asian T2DM group. (a) Linear regression analysis of total cholesterol and TL in T2DM (clear triangles); (b) linear regression analysis of total cholesterol and TL in T2DM by gender (male: dark squares and solid line, females: open squares and dotted line).

**Figure 3:**
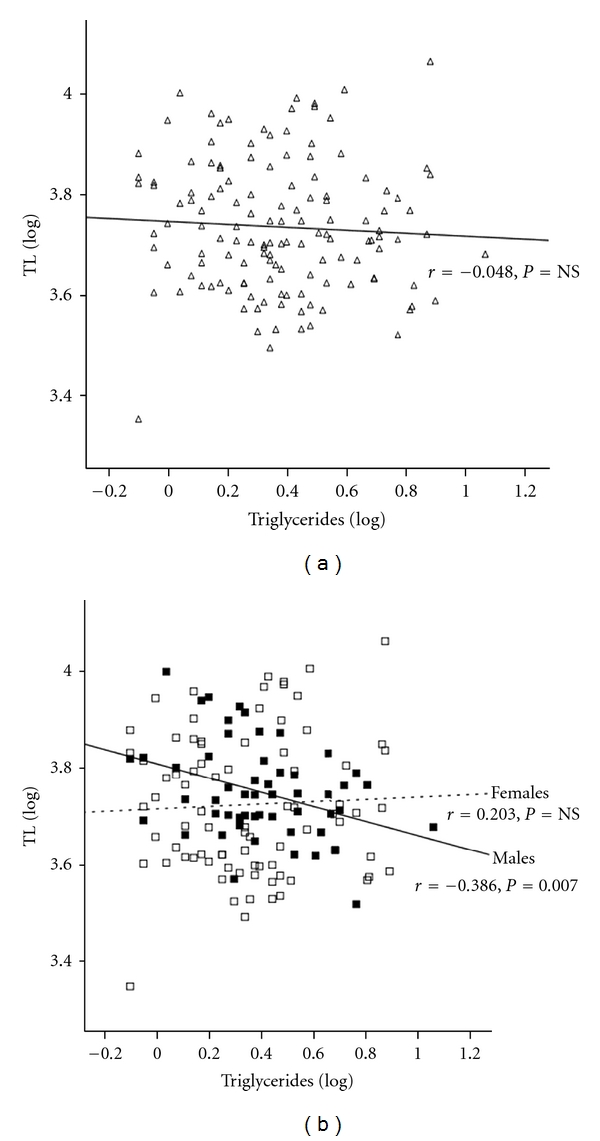
The relationship between triglycerides and TL in South Asian T2DM group. (a) Linear regression analysis of triglycerides and TL in T2DM (clear triangles); (b) linear regression analysis of triglycerides and TL in T2DM by gender (male: dark squares and solid line, females: open squares and dotted line).

**Figure 4:**
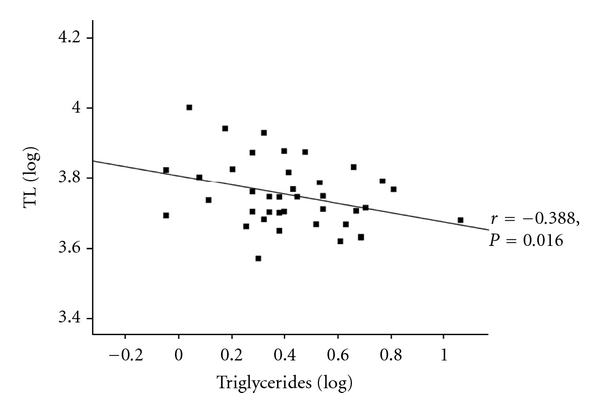
TL in obese male T2DM subjects (BMI: 25–34.9 kg/m^2^) is inversely correlated with triglycerides (*N* = 39).

Given that T2DM was associated with a reduction in TL in South Asians, further analysis investigated whether altered lipid profile had an effect on TL. Total cholesterol, LDL-cholesterol, and triglycerides showed no gender differences (Table 1), but HDL-cholesterol was significantly higher in females compared to males (*P* = 0.0001Table 1). There was no relationship overall between the lipid profiles for the T2DM group (Figures 2(a) and 3(a), data not shown, resp.), but when the cohort was stratified by gender, total cholesterol was inversely associated with TL in T2DM males, even after adjusting for age (*r* = −0.414, *P* = 0.001; age adjusted: *r* = −0.419, *P* = 0.0018). Triglycerides showed a similar pattern, demonstrating an inverse association with age-adjusted TL of T2DM males (*r* = −0.355, *P* = 0.008; age adjusted: *r* = −0.386, *P* = 0.007). These associations were not observed in the female group (Figures 2(b) and 3(b)). Further subgroup analysis, according to BMI, in the male cohort highlighted that in obese subjects triglycerides inversely correlated with TL (*r* = −0.336, *P* = 0.038; age adjusted: *r* = −0.388, *P* = 0.016; *n* = 39; Figure 4). The rest of the associations were noncontributory with no significant effects noted by the use of medication.

## 4. Discussion

In our study, reduced telomere length (TL) observed in T2DM South Asian males was inversely associated with total cholesterol and triglycerides, even after adjusting for confounders. Shortened TL in T2DM subjects has previously been observed in Afro-Caribbeans [14], Arabs [15], and Caucasian subjects [17]. Information regarding TL and T2DM among South Asians is scarce, although in this population shortened TL has been observed for other age-related diseases, especially in vascular diseases (coronary artery disease) and hypertension, and cancer [13, 23, 24]. We observed that non-T2DM male subjects had, on average, shorter TL than non-T2DM females, confirming gender dimorphism which was also observed in other ethnic groups (Europeans, Japanese, as well as Arab children) [25–27]. The nonsignificant associations of TL to other parameters in this study, such as age, BMI and waist circumference, do not supersede prior findings associating TL to age and obesity [15, 27, 28], since it is most likely that the present study's cohort did not reach enough power to elicit significance, or possibly due to the wide range of interindividual variation of TL [27].

The exact mechanism of TL attrition among patients with T2DM is largely explained by insulin resistance, a metabolic condition that promotes reactive oxygen species (ROS) formation and favours a proinflammatory milieu, two factors known to reduce lifespan [29]. One of the most important pathways in the regulation of longevity appears to be endocrine signalling—many mutations that extend lifespan in animal studies involve homologues of insulin and insulin-like growth factor signalling, genes that were observed to extend lifespan by up to 6-fold in model studies [30, 31].

Several hypotheses explain the gender difference in TL amongst non-T2DM subjects in this study. First is the higher stress exposure for males and the protective effect of oestrogen in females. Males are more vulnerable to acute and chronic stress in animal models, as well as in lower occupational class workers, compared with females who are more sensitive to stress but nevertheless more resilient [32, 33]. The presence of chronic psychological stress in modern-day adults is, in part, related to a fast-paced modern lifestyle, which significantly contributes to obesity and dysmetabolic syndrome-related ageing, as it can lead to overeating. Furthermore, coelevation of cortisol and insulin causes accumulation of visceral fat over time, which harbours a biochemical environment conducive to several ageing mechanisms that lead to cell senescence [28, 34]. Benetos and colleagues further explained that at any given chronological age, biological ageing is more advanced in men compared to women, since only in men was TL observed to influence pulse pressure, an index of arterial ageing, hence the conclusion that arterial ageing is modified by gender [35]. In addition, oestrogen deficiency has been shown, in animal models, to inhibit telomerase and, ultimately, lead to telomere shortening [36, 37]. Considering that most, if not all, T2DM women involved in this study were postmenopausal, the protective effects of oestrogen are lost and therefore women, at this stage, tend to catch up with men in the expression of age-related diseases, including telomere attrition.

The significant inverse association of TL with several key lipid parameters (total cholesterol and triglycerides) in South Asian men strengthens the premise that biological ageing is heightened in males, as compared with females of the same chronological age [35]. Differences in triglycerides and HDL-cholesterol between South Asian men and women have also been documented [38], with higher CHD risk for South Asian men as compared to African-Caribbean, white Europeans, and African and Hispanic Americans [39, 40]. The increased predisposition of South Asians to age-related chronic diseases is attributed not only to genetic susceptibility, but also to differences in fat deposition, high physical inactivity and differences in diet [40–42].

The atherogenic properties of elevated cholesterol and triglycerides confer repeated mechanical, hemodynamic, and/or immunological injury and, as such, may cause augmented cell turnover and increased production of ROS in certain cells [28]. The link between cholesterol and TL may be secondary to increased cell damage and turnover, which in turn amplifies cell ageing by bringing cells to their maximum replicative capacity—translating to shortened TL. This could also tie in with age-related innate immune pathway activation in adipose tissue and its link with subclinical chronic inflammation [15].

This study has several limitations. Causality cannot be inferred from this work due to the cross-sectional nature of the study. Furthermore, while most of our findings are confirmatory, it is noteworthy that South Asians are unique compared to other ethnicities, as several factors that include gender discrepancy and metabolic parameters (such as total cholesterol and triglycerides) appear to play a role in the complex maintenance of TL. These observations were either noted in both genders in other ethnic groups or not at all.

In summary, male South Asians with T2DM have shorter TL when compared with their non-T2DM counterparts, and this correlates with triglycerides and cholesterol levels in the same subgroup. This study highlights enhanced biological ageing among South Asian T2DM men, which appears to be tracked by changes in lipids and BMI. This suggests that raised lipids and BMI could directly contribute to premature ageing; however, this possibility needs further study.

## Figures and Tables

**Table 1:** General characteristics of the T2DM group (UKADS).

		United Kingdom Asian Diabetes study		
	All	Females	Males	*P* value
*N*	142	86	56	
Age (years)	53.6 ± 5.8	53.8 ± 6.1	53.2 ± 5.5	NS
BMI (kg/m^2^)	28.4 ± 4.9	30.1 ± 5.1	26.4 ± 3.8	NS
Glucose (mmol/L)	5.6 ± 0.5	5.3 ± 0.6	5.9 ± 0.7	NS
Insulin (*μ*U/mL)	29.9 ± 2.3	30.3 ± 3.4	29.4 ± 3.0	NS
Total cholesterol (mmol/L)	4.7 ± 0.1	4.8 ± 0.2	4.7 ± 0.1	NS
HDL-cholesterol (mmol/L)	1.1 ± 0.02	1.2 ± 0.03	1.05 ± 0.03	0.0001
LDL-cholesterol (mmol/L)	2.4 ± 0.10	2.4 ± 0.1	2.5 ± 0.2	NS
Triglycerides (mmol/L)	2.8 ± 0.2	2.7 ± 0.2	2.95 ± 0.2	NS
Waist circumference (cm)	101.7 ± 12.3	97.2 ± 12.7	105.8 ± 10.4	0.00002
Systolic BP (mmHg)	136 ± 20.0	135 ± 16.0	137 ± 22.0	NS
Diastolic BP (mmHg)	83 ± 10.0	83 ± 10.0	83 ± 11.0	NS
CHD risk (at 10 years %)	13.3 ± 6.0	14.5 ± 5.4	12.1 ± 6.1	NS

Note: NS: nonsignificant; CHD: cardiovascular heart disease.

**Table 2:** General characteristics of non-T2DM subjects (WHSS).

		Wandsworth Heart and Stroke study		
	All	Females	Males	*P* value
*N*	76	33	43	
Age (years)	49.81 ± 5.23	49.67 ± 5.27	49.93 ± 5.26	NS
BMI (kg/m^2^)	25.74 ± 3.61	27.53 ± 3.90	24.38 ± 2.67	NS

Note: NS, nonsignificant.
